# A pooled analysis of pancreatic resection for metastatic renal cell carcinoma

**DOI:** 10.3389/fonc.2024.1442256

**Published:** 2024-11-15

**Authors:** Yanming Zhou, Xiao Wang, Shi Chen, Shijie Wang

**Affiliations:** Department of Surgery, First Affiliated Hospital of Xiamen University, Xiamen, China

**Keywords:** renal cell carcinoma, metastases, resection, pancreas, survival

## Abstract

**Background:**

Pancreatic metastasis from renal cell carcinoma (PMRCC) is unusual and there is no consensus on its treatment. The present study aims to evaluate the clinical outcomes of surgical resection for PMRCC.

**Methods:**

PubMed and Web of Science were searched for Eligible studies from January 1980 to January 2024. Individual-patient data were pooled.

**Results:**

A total of 436 participants were identified. The morbidity and 90-day mortality were 38.1% and 3.4%, respectively. Post-pancreatectomy recurrence occurred in 44.1% of the patients. The overall median survival was 116 months, with a 3-, 5- and 10-year survival rate of 85.3%, 76.6%, and 46.5% respectively. On univariate analysis, repeat metastasectomy was associated with a significantly better prognosis (*P* =0.003).

**Conclusion:**

These data suggest that surgical resection is a safe and effective therapeutic option for PMRCC. Repeat metastasectomy is positively suggested for recurrent disease provided all metastases can be removed curatively.

**Systematic review registration:**

https://www.crd.york.ac.uk/prospero/, identifier CRD42024525218.

## Introduction

Renal cell carcinoma (RCC) is one of the most common urinary cancers, causing 179,368 deaths in 2020 worldwide (1), and half of these patients either had synchronous metastases at presentation or developed metachronous metastases after nephrectomy (2). RCC commonly spreads to the lung, bone, liver, and brain, and metastasis to the pancreas is rare, with the reported incidence of lower than 1% (3). Publications on surgical intervention of pancreatic metastases from RCC (PMRCC) are mostly in the form of case reports and small series (4–10), making it difficult to draw a definitive conclusion about their therapeutic outcomes. This study aims to evaluate the clinical value of pancreatectomy in the treatment of PMRCC based on a pooled analysis of individual-patient data derived from previous publications in the literature.

## Methods

The present study was registered in PROSPERO (registry number:CRD42024525218) and conducted in adherence to the Preferred Reporting Items for Systematic Reviews and Meta-Analyses (PRISMA) (11). Two authors independently performed literature search, data extraction, and assessment of the methodological quality of the included studies. Any disagreement was resolved by discussion and consensus.

### Search strategy

The electronic search was carried out using PubMed and Web of Science from January 1980 to January 2024. Search terms were: pancreatic metastasis, renal cell carcinoma, and resection. The references of all retrieved articles were screened manually for additional publications.

### Inclusion and exclusion criteria

Included studies were case reports and case series that reported the clinical outcomes of PMRCC patients who underwent pancreatectomy with curative intent. Non-English-language articles, abstracts, reviews without original data, overlaps, and reports with uncertain follow-up data or without presenting individual data were excluded.

### Data extraction

Data on patient demographics, presenting symptoms, intervals from nephrectomy to pancreatic metastasis or disease-free interval (DFI), surgical outcomes, pathological findings, and long-term survival were extracted. Late recurrence was defined as PMRCC that developed in patients with a 120-month DFI since the initial nephrectomy (12). Overall survival (OS) was calculated from the time of pancreatic metastasectomy to the time of death or last follow-up. Pancreatic fistulas were graded according to the International Study Group on Pancreatic Fistula (ISGPF) (13). Postoperative mortality was defined as death occurring within the 90-day postoperative course

The quality of the included studies was assessed using the Oxford Centre for Evidence-Based Medicine scoring system (14).

### Statistical analysis

Descriptive statistics were applied for clinicopathologic characteristics parameters. Continuous variables are expressed as median with range, and categorical variables are expressed as frequencies and percentages. Missing values were not imputed. OS was generated by the Kaplan-Meier method and compared by the log-rank test. Prognostic factors affecting survival were identified using logistic regression models and presented as hazard ratio (HR) with 95% confidence intervals (CI). Statistics were performed via SPSS version 18.0 (SPSS Inc, Chicago, Illinois, USA). A 2-sided *P* < 0.05.

## Results

**Figure 1:**
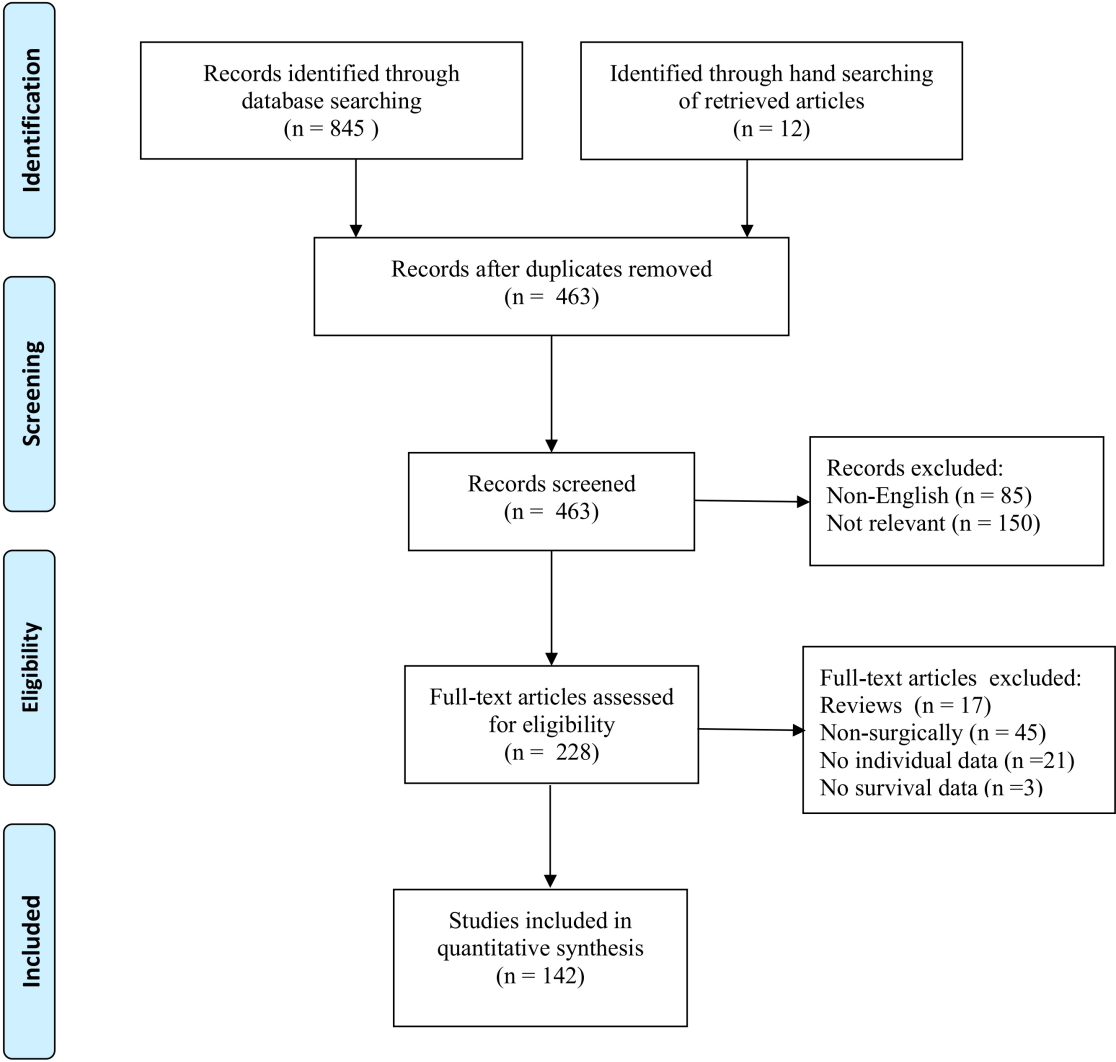
PRISMA diagram of systematic literature review.

The systematic search identified 142 studies (Appendix A) eligible for inclusion, reporting on 436 patients who underwent pancreatectomy for PMRCC (**Figure 1**). All studies were retrospectively designed and therefore graded as low evidence of level 4.

### Patient characteristics

The median age at the time of pancreatic resection was 64 (range 33–88) years with a slight male predominance (51.1%) (**Table 1**). More than half of the subjects (59.6%) were asymptomatic at the time of diagnosis. The most common presenting symptom was abdominal or back pain (45.4%), followed by jaundice (21%), weight loss (21%), and gastrointestinal bleeding (20%). The primary RCC originated from the left kidney in 53.7% patients, right kidney in 44.4% patients, and both kidneys in 1.9% patients.

**Table 1:** Patient clinical and pathologic characteristics.

Parameters	Value
Sex (n = 413), no. (%)	
Male	211 (51.1)
Female	202 (48.9)
Age at presentation of metastasis (n = 409)	
Median (range), years	64 (33-85)
Primary tumor characteristics	
Side (n = 324), no. (%)	
Right kidney	144 (44.4)
Left kidney	174 (53.7)
Bilateral	6 (1.9)
Stage (n = 106), no. (%)	
pT1	26 (24.6)
pT2	40 (37.7)
pT3	40 (37.7)
Lymph node involvement (n = 97), no. (%)	
Positive	5 (5.2)
Extrapancreatic metastases (n = 432), no. (%)	
Present	92 (21.3)
Before pancreatic lesion	46
Before and synchronously	12
Synchronously	34
Pancreatic metastases characteristics	
Presentation (n = 302), no. (%)	
Symptomatic	122 (40.4)
Abdominal or back pain	56
Jaundice	25
Weight loss	25
Gastrointestinal bleeding	24
Timing of metastases (n = 436), no. (%)	
Synchronous	23 (5.3)
Metachronous	413 (94.7)
Metastatic interval (n = 423), no. (%)	
< 12 months	34 (8.0)
12-59 months	79 (18.7)
60-119 months	122 (28.8)
≥120 months	188 (44.4)
Metastatic location (n= 333), no. (%)	
Head	116 (34.8)
Uncinate	3 (0.9)
Body	51 (15.3)
Tail	66 (19.8)
Multi-regions	107 (32.1)
Type of pancreatectomy (n= 436), no. (%)	
Pancreaticoduodenectomy	143 (32.8)
Distal pancreatectomy	162 (37.1)
Total pancreatectomy	80 (18.3)
Other procedures	51 (11.7)
Enucleation	15
Middle-preserving pancreatectomy	3
Middle pancreatectomy	8
Middle pancreatectomy + enucleation	2
Subtotal pancreatectomy	2
Pancreaticoduodenectomy + enucleation	2
Distal pancreatectomy + enucleation	7
DPPHR	7
DPPHR + distal pancreatectomy	1
Unspecified	4
Morbidity (n= 226), no. (%)	
Present	86 (38.1)
Pancreatic fistula (n= 222), no. (%)	
Present	33 (14.9)
Mortality (n= 436), no. (%)	
Present	15 (3.4)
Tumor size at diagnosis (n = 254)	
Median (range), cm	3.0 (0.8-15)
Tumor number (n= 342), no. (%)	
Solitary	211 (61.7)
Lymph node involvement (n= 189), no. (%)	
Positive	13 (6.8)
Surgical margin (n= 281), no. (%)	
Positive	11 (3.9)
Recurrence (n= 376), no. (%)	
Present	166 (44.1)
Lung	54
Remnant pancreas	35
Liver	32
bone	13
Brain	15
Kidney	13
Adrenal gland	6
Thyroid	9
Lymph node	10
Peritoneum	9
Other sites #	16
Unspecified	15

#(muscle 2; retroperitoneum 2; forearm 4; parotid 2; chest wall 1; omentum1;

mesentery 1; heart 1; periprostatic tissue 1; stomach 1)

DPPHR, duodenum-preserving pancreatic head resection

RCC metastases to the pancreas were synchronous in 5.3% patients and metachronous in 94.7% patients. The median DFI was 108 months (range 0-432) and 44.4% patients developed late recurrences. Lesions were localized at the pancreatic head in 34.8%, at the body in 15.3%, at the tail in 19.8%, and at the multi-pancreatic regions in 32.1% of patients.

Of the 432 patients with reported information, 92 (21.3%) had at least one extra-pancreatic metastatic lesion before and/or synchronous pancreatic metastasis involving the lung (31), thyroid (18), kidney (17), liver (13), adrenal gland (8), brain (7), lymph nodes (4), retroperitoneum (3), breast (2), ileum (2), parotid gland (2), peritoneum (2), chest wall (1), bone (1), falciform ligament (1), hip (1), arm (1), diaphragm(1), deltoid muscle (1), subcutaneous tissue (2), shoulder (1), spleen (1), trachea (1), tongue (1), scapula (1), abdominal wall (1), buttock (1), oral cavity (1), and hamstrings (1). Treatments were metastasectomy (81, 88.0%), radiation (2, 2.2%), systemic therapy (3, 3.3%), and unspecified (6, 6.5%).

### Surgical outcomes

Of the 436 patients, 80 (18.3%) patients underwent total pancreatectomy, and the other 356 patients underwent partial pancreatectomy. The postoperative morbidity rate was 38.1%. Pancreatic fistula occurred in 33 (14.9%) of the 222 reported patients, and only two (6%) of them had grade C pancreatic fistula. Of the 436 patients, 15 patients (3.4%) died during the postoperative course due to multiorgan failure (3), malignant hyperpyrexia (1), heart failure (2), myocardial infarction (1), hemoperitoneum arising from splenic artery pseudoaneurysm rupture (1), liver failure (1), disease progression (1), pulmonary embolism (1), and unspecified reasons (4).

Histopathological examination revealed that the median tumor size was 3.0 (range 0.8-15) cm, and solitary metastases were identified in 61.7% patients. The overall incidence of nodal involvement was 6.8%, and rate of tumor-positive resection margins was 3.9%.

### Long-term outcomes

After excluding perioperative mortality, long-term outcomes were assessed in 421 patients. Recurrent disease occurred in 166 (44.1%) of 376 patients with provided information. The most common recurrence site was the lung, followed by the remnant pancreas and liver. Of them, 49 patients underwent 62 repeat metastasectomies. Recurrence sites in the repeat surgery group were the pancreas (22), lung (10), thyroid (6), liver (5), kidney (4), forearm (4), adrenal gland (3), lymph node (2), bone (2), parotid (1), musculus obturatorius externus (2), and periprostatic tissue (1).

**Figure 2:**
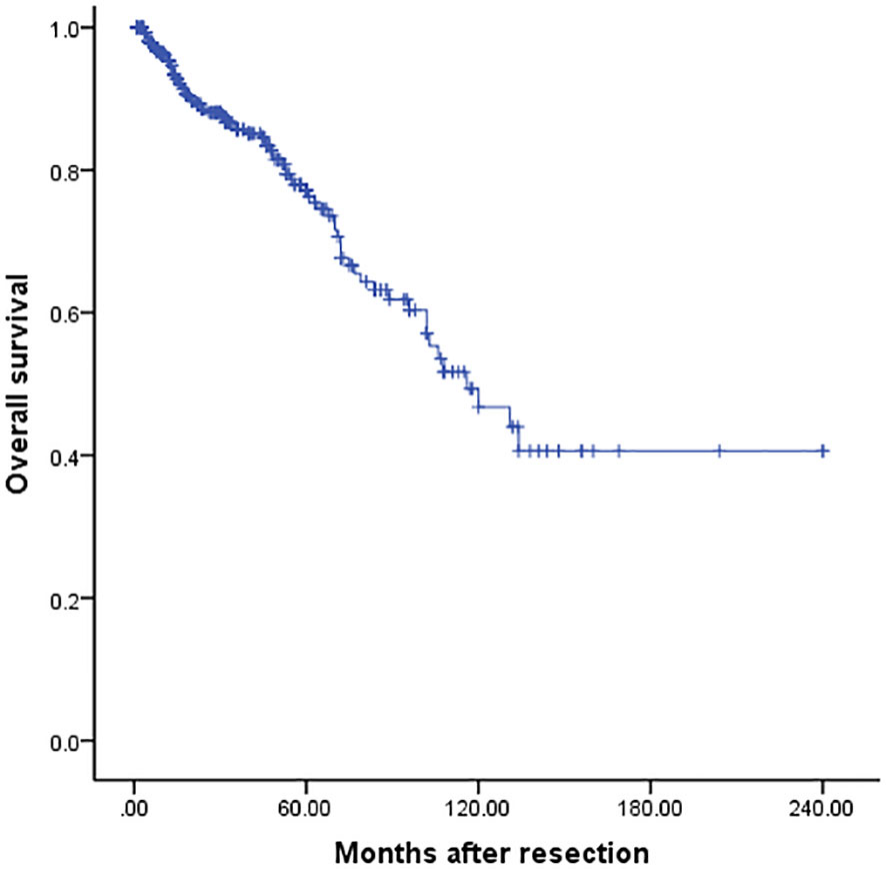
Overall survival curve following resection.

For the entire cohort, the median survival was 116 months, and the 3-, 5- and 10-year OS was 85.3%, 76.6%, and 46.5% respectively (**Figure 2**). Univariate analysis revealed that only repeat metastasectomy was associated with a significantly better prognosis (**Table 2**). Multivariable analysis was not performed because only one variable had a value of *P* < 0.05 in univariable analysis.

**Table 2:** Univariable analysis of factors associated with overall survival.

Characteristics	HR (95% CI)	*P*
Sex		
Female/male	0.921 (0.584-1.453)	0.726
Age, years		
≥ 60/< 60	0.968 (0.601-1.558)	0.894
Site of primary lesion		
Left/right	0.823 (0.453-1.496)	0.523
Stage of primary lesion		
pT2-T3/pT1	1.633 (0.204-13.536)	0.635
Symptoms		
Yes/no	0.761 (0.436-1.327)	0.336
Extrapancreatic metastases		
Yes/no	1.013 (0.604-1.699)	0.960
Metastatic interval, months		
≥ 36/< 36	1.302 (0.751-2.258)	0.347
≥ 120/< 120	0.759(0.483-1.192)	0.231
Metastases size, cm		
≥ 4/< 4	1.091 (0.554-2.150)	0.801
Metastases number		
Multiple/single	1.439 (0.798-2.596)	0.226
Lymph node metastasis		
Positive/negative	1.445 (0.343-6.097)	0.615
Surgical margin		
Positive/negative	1.506 (0.468-4.847)	0.492
Type of pancreatectomy		
Partial/total	1.335 (0.722-2.469)	0.356
Repeat metastasectomy		
Yes/no	0.241 (0.095-0.610)	0.003

HR, Hazard ratio; CI, Confidence interval.

## Discussion

To the best of our knowledge, this review of results in 436 patients constitutes the largest collective series on surgery for PMRCC yet reported. In comparison to a systematic review on this subject published in 2009 by Tanis et al. (40), a significant proportion (49.3%) of patients included in our report were reported in studies after 2010, indicating that our study is closer to the modern practice. The 5- and 10-year OS rates were 76.6% and 46.5% respectively, confirming the value of pancreatic resection for PMRCC in contributing to favorable long-term outcomes. There have been concerns regarding the safety of pancreatic resection, especially metachronous resection, where intra-abdominal adhesions may be present by the previous operation. However, the present study demonstrated that such operations can be undertaken safely with low mortality and acceptable morbidity, reflecting the improvements in surgical techniques and perioperative patient care in the field of pancreatic surgery over the last several decades.

**Table 3** summarizes the results of published series that were excluded from the analysis because individual patient data could not be extrapolated (8–10, 15–39). The median overall morbidity and mortality were 44.3% (range16–67%) and 2.4% (range 0–14.3%) respectively. During a median follow-up of 56.7 (range 31–104) months, 54.3% patients (range 35–100%) developed recurrences. The median survival since pancreatectomy was 75 (range 30.5–147.9) months with a 5-year OS rate of 71.8% (range 38–92.8%). The fact that these studies reported varied prognosis, probably due to the significant clinical heterogeneity of the study groups, rendering meta-analysis of survival outcomes inappropriately. Notable, most of these series contained very small number of patients, making them unable to provide specific data on prognostic factors of survival. In contrast, a pooled analysis of individual patient data from previous reports will increase the statistical power to address this issue.

**Table 3:** Summary of published series excluded from this analysis.

Reference	Country	Year	N	DFI (months)	EPM, %	MTS, (cm)	ST,%	LNM, %	Morbidity, %	Mortality, %	MFu (months)	Rec, %	MOS, (months)	5- yr OS, %
Hiotis (8)	USA	2002	10	–	0	–	–	–	–	10	–	–	57.6	38
Bassi (9)	Italy	2003	17	–	0	3	52.9	0	47.1	0	33	35.3	–	53
Jarufe (10)	UK	2005	7	129.6	–	–	–	–	–	14.3	–	–	30.5	–
Zerbi (15)	Italy	2008	23	144	–	3	65.2	–	47.8	0	31	47.8	–	88
Strobel (16)	Germany	2009	31	123.6	–	3	–	20	–	6.4	–	55	–	67.3
Konstantinidis (17)	USA	2010	20	104.4	30	3	75	25	–	0	36	–	104	61
Facy (18)	France	2013	13	90	38.5	3	61.5	0	–	0	48	50	–	75
Schwar (19)	France	2014	62	120	23	3.5	62.9	27.3	–	6.5	91	63.7	–	63
Tosoian (20)	USA	2014	42	138	21.1	3.8	57.1	5.1	26	4.7	84	–	66	51.8
Benhaim (2)	France	2015	20	130	0	2	65	–	40	5	69	55	–	66
Santoni (22)	Italy	2015	44	–	4.7	–	–	–	–	0	–	67	103	65
Yuasa (23)	Japan	2015	15	–	13.3	2.1a	–	–	16	0	42	–	–	72
Fikatas (24)	Germany	2016	18	122	22.2	–	–	–	22.2	0	49	38.9	–	71.4
Rückert (25)	Germany	2016	40	125.4	32.5	–	45	22.7	–	7.5	–	–	147.9a	78
Madkhali (26)	Korea	2018	17	–	35.3	2.3	–	0	–	–	–	100	–	50
Anderson (27)	USA	2020	29	96	0	2.4a	51.7	–	44.8	0	64.7	55.1	–	–
Brozzetti (28)	Italy	2020	26	156	53.8	–	30.7	13.6	53.8	0	104	46.1	–	76.9
Di Franco (29)	Italy	2020	21	83	0	2.7	38.1	23.8	28.6	0	76.7	42.8	75	71.6
Milanetto (30)	Italy	2020	39	84	17.9	2.5	46.1	12.8	38.5	2.6	–	52.8	134	79
Shin (31)	Korea	2021	66b	0	–	–	–	–	–	–	–	–	40.3	–
			132c	63	–	–	–	–	–	–	–	41.7	53.7	–
Blanco-Fernández (32)	Spain	2022	116	87.4a	23.3	2.4	74.1	3.4	60.9	3.4	43	55.3	105	83
Kinoshita (33)	Japan	2023	23	–	–	–	–	–	–	0	–	–	–	78.6
Moletta (34)	Italy	2023	16	–	–	–	–	0	43.7	6.2	–	60	119	–
Riemenschneider (35)	Denmark	2023	25	–	36	–	–	–	36	4	69.6	62.5	134.8	83.6
Al-Madhi (36)	Germany	2024	17	154	58.8	–	58.8	0	64.7	5.9	43	37.5	–	72
Boubaddi (37)	France	2024	42	121	42.8	2.3	54.8	–	66.7	2.4	76	70.7	–	92.8
Hajibandeh (38)	UK	2024	18	58	0	3.7a	–	–	61.1	0	–	–	64	55.6
Lee (39)	Korea	2024	56	–	–	1.6	–	1.8	–	–	–	53.6	43.2	87.5

DFI, disease-free interval; EPM, extrapancreatic metastases; MTS, median tumor size; ST, solitary tumor; LNM, lymph node metastasis;

MFu, median follow-up; MOS, median overall survival; Rec, recurrence.

a mean; b synchronous metastasis; c metachronous metastasis.

About 21.3% of the patients reported in this study had additional extra-pancreatic lesions, and most of these lesions were resected. The survival time of these patients was not significantly different from that in the other patients (*P* = 0.960) (**Table 2**), which is consistent with the result reported by Tanis et al. (40). Therefore, it is reasonable to offer pancreatic surgery to patients with extra-pancreatic lesions as long as the metastases have been managed curatively.

As demonstrated in the current study, PMRCC presented a long median DFI of 108 months, and late recurrences were not an uncommon phenomenon. Thus, a lifelong surveillance is necessary for patients with a history of RCC. Some authors noted that a prolonged DFI (≥ 36 months) was associated with a better prognosis probably due to a less aggressive biological behavior of slow tumor growth (16), but this relationship was not validated in the current study.

Consistent with prior studies (30, 32), neither multiplicity nor size of PMRCC was found to confer a significant effect on patient survival. It seems that surgical treatment is indicated for all PMRCC cases provided it is technically feasible.

The extent of pancreatectomy remains a controversy in the treatment of PMRCC, especially in the setting of multifocal disease (9, 32, 37). Theoretically, total pancreatectomy has advantages of complete removal of occult micrometastases and is thought to reduce the risk of pancreatic recurrences. However, this extensive surgical resection may lead to major metabolic problems, culminating in the poor quality of life. It was found in this study that partial pancreatectomy did not impair survival outcomes, suggesting that such pancreas-sparing surgical procedures should be encouraged as an initial treatment for PMRCC. In order to achieve radicality, thorough exploration of the pancreatic parenchyma by palpation and/or ultrasound is required (18). Nevertheless, for diseases involving the entire pancreas, total pancreatectomy is indicated.

We found that lymph node involvement in PMRCC was rare and therefore had little prognostic significance, which is also supported by data from most previously published series (**Table 3**). As many surgeons were hesitant to perform routine lymphadenectomy for PMRCC in clinical practice, most patients (56.7%) presenting insufficient lymph node information were included in this pooled analysis. Nevertheless, from an oncological perspective, we believe that lymphadenectomy may provide staging information for personalized adjuvant therapy.

Nevertheless, it should be emphasized that recurrence after resection is common, clearly exceeding 40%. The problem arises in this condition as to whether a repeat metastasectomy could be indicated. However, little information exists about this issue to date. A striking finding in this study was that repeat metastasectomy confer substantial survival benefits, highlighting the necessity for surgical resection of relapse provided all metastases can be complete eradicated.

Although encouraging long-term survival outcomes have been seen after surgical management, the question of whether this aggressive approach is a preferable option for PMRCC remains in doubt in the light of recent advances in systemic therapy, including tyrosine kinase inhibitors (TKI) and immune checkpoint inhibitors (ICI). Santoni and colleagues concluded that surgical resection and TKI therapy could provide similar median OS (103 vs. 86 months; *P* = 0.201) (22). However, their study population was not large enough and there may be a bias in patient selection.

The main limitation of the present review is its retrospective design with inherited risk of information loss. Consequently, patients were not stratified by the Memorial Sloan-Kettering scoring system, and therefore no analysis regarding cancer-specific survival and disease-free survival could be carried out. Similarly, in the modern era of multidisciplinary strategies for metastatic RCC (41), we are unable to evaluate the role of pancreatic metastasectomy in conjunction with targeted and immune therapy, and therefore further investigation is required in future.

## Conclusion

In summary, surgical resection is a safe and effective therapeutic option for PMRCC. In addition, recurrent disease should be treated by repeat metastasectomy provided all metastases can be removed curatively.

## Data Availability

The original contributions presented in the study are included in the article/supplementary material. Further inquiries can be directed to the corresponding author.
